# Hyaluronic Acid: A Key Ingredient in the Therapy of Inflammation

**DOI:** 10.3390/biom11101518

**Published:** 2021-10-15

**Authors:** Andreia Marinho, Cláudia Nunes, Salette Reis

**Affiliations:** LAQV, REQUIMTE, Departamento de Ciências Químicas, Faculdade de Farmácia da Universidade do Porto, 4050-313 Porto, Portugal; aierdnamarinho@gmail.com (A.M.); shreis@ff.up.pt (S.R.)

**Keywords:** hyaluronic acid, natural polymer, inflammatory diseases, drug delivery

## Abstract

Hyaluronic acid (HA) is a natural polymer, produced endogenously by the human body, which has unique physicochemical and biological properties, exhibiting desirable biocompatibility and biodegradability. Therefore, it has been widely studied for possible applications in the area of inflammatory diseases. Although exogenous HA has been described as unable to restore or replace the properties and activities of endogenous HA, it can still provide satisfactory pain relief. This review aims to discuss the advances that have been achieved in the treatment of inflammatory diseases using hyaluronic acid as a key ingredient, essentially focusing on studies carried out between the years 2017 and 2021.

## 1. Introduction

**Figure 1:**
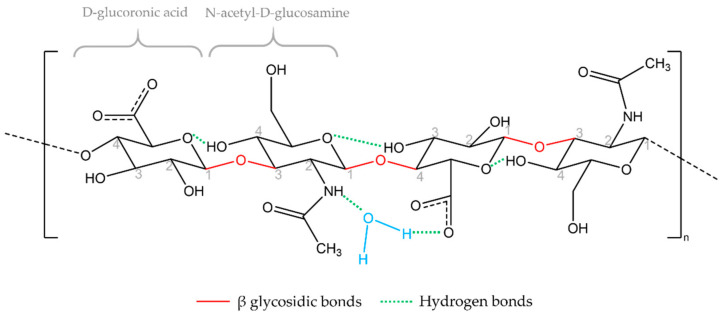
Chemical structure of hyaluronic acid (HA), where the disaccharide units that compose it are shown: D-glucuronic acid and N-acetyl-D-glucosamine. Green is shown by hydrogen bonding, including water bonding, and red signifies glycosidic bonds that occur in the molecule.

Hyaluronic acid (HA) is a natural, unbranched polymer that belongs to a group of glycosaminoglycan heteropolysaccharides (GAGs), which are the main components of the extracellular matrix (ECM) [1,2]. It is a molecule with a simple chemical structure (Figure 1), of a polar (hydrophilic) nature, composed of repeated disaccharide units of D-glucuronic acid and N-acetyl-D-glucosamine, linked by alternating β-1,3 and β-1,4 glycosidic bonds [3,4,5,6,7,8]. In its native form, HA appears as a very long polymer, called high-molecular weight HA (HMWHA) [2]. However, under certain conditions, the molecule can be broken down into small fragments, called low-molecular weight HA (LMWHA) [2]. HMWHA, usually greater than 1000 kDa, is present in intact tissues and is antiangiogenic and immunosuppressive, while smaller polymers indicate signs of distress and are potent inducers of inflammation and angiogenesis [6,9,10].

The literature reports some works, namely, the reviews by Fallacarra et al. [1] and Litwiniuk et al. [2], where the structural and biological properties of HA are reported and where medical, pharmaceutical, and cosmetic applications are used, including the role of HA in inflammation and tissue regeneration. This review, in addition to surveying the physicochemical and biological properties (biosynthesis, degradation, hydration, mechanism of action, interaction with membrane receptors, etc.) intends to show the wide potential of HA in therapy for inflammatory diseases, approaching topics including its benefits in pathologies of the joints, intestines, lungs, heart, etc.

### 1.1. Biosynthesis

**Figure 2:**
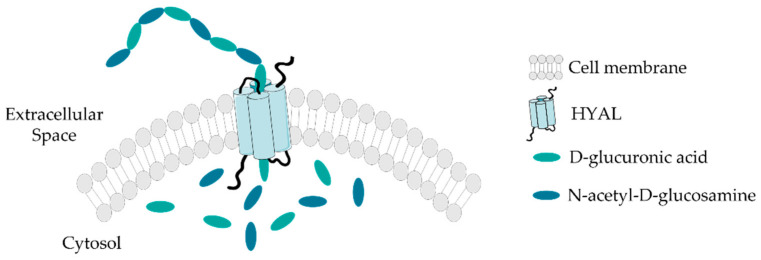
Schematic representation of the synthesis of HA by hyaluronan synthases (HAS-1, HAS-2 and HAS-3) where the placement of HA in the extracellular space is evidenced.

Most cells in the human body are able to synthesize HA during certain processes in their cell cycle [4,11], although mesenchymal cells are believed to be the predominant source of HA [8,12]. HA differs from other GAGs because it is not sulphated and is not synthesized by Golgi enzymes in association with proteins; it is produced in the inner face of the plasma membrane, without any covalent binding to a protein nucleus, by hyaluronan synthases (HAS-1, HAS -2 and HAS-3) [1,13,14,15]. These three proteins place the HA molecules in the extracellular space through pore-like structures, along the length of the polymeric chain [2,8,16,17], repeatedly adding D-glucuronic acid and N-acetyl-D-glucosamine (Figure 2), giving rise to molecules of different sizes [11,18]. This unique synthesis mechanism, which allows the molecule to be secreted during its production, is essential; otherwise, due to its enormous size, the cells would be destroyed [19]. Incorrect deregulation of the expression of HAS genes results in abnormal HA production and, therefore, an increased risk of pathological events, altered cellular responses to injuries and aberrant biological processes [1,20]. It has also been established that growth factors, such as epidermal growth factor (EGF) or keratinocyte factor, increase the rate of HA synthesis [17].

As previously reported, mesenchymal cells are believed to be the predominant source of HA; however, some studies report that other cell types are capable of producing large amounts of HA. An example of this is the study described by Sapudom et al., where it is verified that activated fibroblasts are capable of producing large amounts of HA. This study was carried out using 3D collagen matrices, which mimic the ECM of a tumor microenvironment, where the normal dermal fibroblast and activated fibroblasts were incorporated. In addition to verifying that the activated fibroblasts produce a large amount of HA, it was possible to verify that the average weight of HA produced by these cells was around 480 kDa, and that this was produced via HAS-2 [21].

### 1.2. Degradation

**Figure 3:**
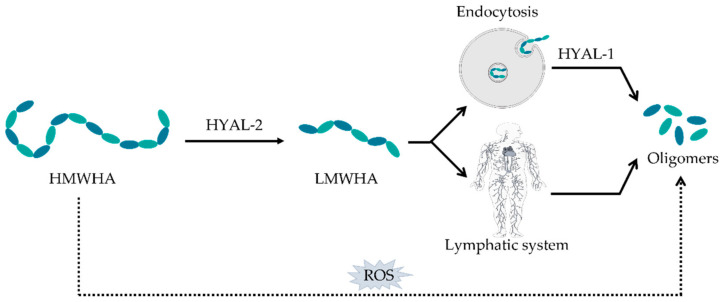
Representative scheme of enzymatic and ROS degradation of HA.

The degradation of HA (Figure 3) in the human body is carried out by two distinct mechanisms: one is specific, mediated by enzymes (hyaluronidases, HYALs), while the other is non-specific, determined by oxidative damage due to reactive oxygen species (ROS) [1,22]. HYALs are a family of enzymes that degrade HA by cleaving the β-1,4 glycosidic bonds, fracturing the large molecule into smaller pieces before degrading it [7,23,24]. First, HYAL-2 degrades HA into molecular weight fragments reaching 20 kDa [2,25,26,27]. These HA molecules are subsequently endocytosed and delivered to lysosomes, where further digestion is performed by HYAL-1 [2,25,26,27]. By catalyzing the hydrolysis of HA, HYALs decrease the viscosity of HA, thereby increasing tissue permeability [28]. HMWHA can also be naturally degraded in the body by mass-produced ROS during the inflammatory response, tissue damage and tumorigenesis [1,26]. The depolymerization of HA occurs through mechanisms that are dependent on ROS, which always involve the splitting of glycosidic bonds [1,26]. Together, HYALs and ROS degrade approximately 30% of the 15 g of HA that is locally present in the human body [26,29]. The remaining 70% are systematically catabolized: HA is mainly transported by the lymph to the lymph nodes, where it is internalized and catabolized by the endothelial cells of the lymphatic vessels [1,26,29,30]. In addition, a small amount of HA is transported into the bloodstream and degraded by the liver’s endothelial cells [1,26,29,30].

It is believed that it is difficult for the body to absorb a polysaccharide [31]. HA is not absorbed by the body as a high-MW polymer after ingestion [31]. An in vitro assay using Caco-2 cells revealed that HA with an MW greater than 100 kDa is rarely absorbed. On the contrary, the amount of HA absorbed by the cells increases, as the MW of the HA decreases to 70, 20 or 5 kDa [31]. There are reports that HA is decomposed into two- to six-membered polysaccharides by enteric bacteria, and these polysaccharides are then partially absorbed in the body by the small intestine, and can move into the joints and other tissues [31]. Accounting for these degradation mechanisms, which occur continuously in vivo, it is estimated that the half-life of HA in the skin is 1–2 days, the half-life in the bloodstream about 24 h, in the eye, it is 24–36 h, 1–3 weeks in the cartilage and about 70 days in vitreous humor [1,2,6,32]. The balance between the synthesis and degradation processes of HA plays an essential regulatory role in the human body, as it determines not only the amount of HA, but also its MW [1,2]. It is the MW and circumstances of synthesis or degradation that determine the biological actions [1,2].

HA turnover is finely regulated by enzymatic synthesis and degradation and, when compared to other ECM components, this turnover is relatively fast [1,33,34,35]. Of the estimated 15 g in humans, about a third is reversed every day in situ [26,33,34,35,36]. Although why this metabolism is so active in the body is unknown, the intrinsic capacity of HA to act as an eliminator of ROS is possibly an advantage in its rapid turnover [33]. During the inflammatory process, this turnover is disturbed, leading to the accumulation of the fragments associated with the spread of the inflammatory response in the extracellular spaces [27].

### 1.3. Hydration

Due to its molecular structure at a neutral pH, HA attracts water molecules and can contain up to 10,000 times its weight in water [9,26,29,33,35]. As shown in Figure 1, water molecules are linked by hydrogen bonding, which stabilizes the molecule’s structure. In aqueous solution, due to hydrophobic interactions and intermolecular hydrogen bonding, polymeric chains aggregate, with the formation of loose and elastic matrices, which facilitate cell migration [1,13,16,29,33,37]. The organization of the matrices that are formed varies according to the type of tissue and the cellular microenvironment [38]. Within these HA matrices, pores appear that allow small molecules to diffuse freely, while macromolecules are partially excluded [16,39]. However, HA matrices, by nature, are very dynamic so they move constantly in solution, leading to continuous changes in pore size [16]. These changes mean that even the macromolecules have the potential to cross the matrix [16].

## 2. Biological Functions

The fact that HA is produced by practically all types of cell means that, in normal biological states, HA has multiple fundamental biological functions [4,8,11,12]. HA can be involved in several cellular interactions (differentiation, proliferation, development and recognition) and biological functions (lubrication, hydration, matrix structure and steric interactions) [6,11]. The viscous gel formed by the HA matrices lubricates the joints and acts as a buffer for the surrounding tissues [40], as well as participating in tissue regeneration and remodelling processes, for example, during the healing process [29]. This molecule has mechanical and dilation properties that can adjust cellular functions, such as adhesion and expansion, and form structures, such as microvilli, which can play an important role in signal transmission [4]. In addition to these properties, HA can alter the local properties of the cell membranes, acting as an external cytoskeleton, and modifying and controlling the shape of the cell [4]. During tissue injury, HA is actively produced, regulating tissue repair and disease processes, such as the activation of inflammatory cells, to initiate an innate response to the injury and regulate the behavior of epithelial cells and fibroblasts [12].

In addition, the anti-inflammatory, immunomodulatory, anti-proliferative, anti-diabetic, anti-aging, wound healing and tissue regeneration, skin repair, and cosmetic properties [3,41] that this molecule presents make it attractive for biomedical applications.

It has already been shown that HA can alter the physical properties of the ECM, including, for example, the viscosity and organization of collagen fibrils. These effects have been attributed to the unique structure of HA, a long, negatively charged, randomly coiled chain spanning an enormous hydrodynamic domain of the collagen fibrils within the ECM [42]. Several studies show that increasing the concentration of HA in the matrix can change the viscoelastic properties of the matrix. The matrix pore can be filled with HA molecules, which leads to a decrease in cell migration as the HA concentration increases. It is also reported that HA degradation by hyaluronidases in the tumor microenvironment triggers tumor progression [42,43,44]. This shows that the presence of HA influences matrix rigidity, and together, they can modulate cell behavior.

## 3. Mechanism of Action

HA performs its biological functions according to two basic mechanisms: (I) it can act as a passive structural molecule and (II) as a signalling molecule [1,40]. The passive mechanism is related to the physicochemical properties of HMWHA [1,40]. Due to its macromolecular size, marked hygroscopicity, and viscoelasticity, HA is able to modulate the tissue hydration, osmotic balance and physical properties of ECM, structuring a hydrated and stable extracellular space, where cells, collagen and elastin fibers, among other ECM components, are firmly maintained [1,40]. When interacting with its binding molecules, HA also acts as a signalling molecule [1,18,45,46]. Depending on its MW, location and specific cell factors (expression of the receptor, signalling pathways and cell cycle), the link between HA and its proteins determines opposing actions: pro- and anti-inflammatory activities, the promotion and inhibition of migration activation and blocking of cell division and differentiation [1,18,45,46].

Furthermore, HA can interact with specific cytokines, and thus modulate immune cell function. One example is interleukin-8 (IL-8), released by fibroblasts, monocytes/macrophages, endothelial and epithelial cells in the presence of inflammation [47]. It is known that HA has a weak binding to IL-8, which means its use as a coating compound is possibly advantageous in the inhibition of unwanted immune response [48]. Although there are studies reporting the interaction between IL-8 and HA, no binding site has been determined. Therefore, it is believed that the interaction between GAGs and IL-8 is not solely an electrostatic process, but that steric interactions as with hydrogen bonds are also crucial to the specificity of the interaction [47]. Furthermore, these interactions are dependent on the sulfation degree of the GAG and the concentration of IL-8 [48], so the introduction of sulfate groups to the HA chain significantly improves its binding to IL-8 [47].

## 4. Interaction with ECM Molecules and Cell Surface Receptors

It is evident that the biological and pharmacological effects of HA are mediated by interactions with certain molecules of ECM and cell surface receptors, the hyaladherins [26,49,50,51]. Interactions with hyaladherins are important in the development of cells and organs, in the response to tissue damage and inflammation, cell migration, formation and resistance to cancer [52]. Within this class of molecules there are three main classes: (I) CD44—cluster determinant 44, (II) RHAMM—Receptor for HA-mediated motility, and (III) ICAM-1—intercellular adhesion molecule 1 [11,53].

In addition to these main receptors, others have been identified for binding to HA, namely: (I) HA-receptor for endocytosis (HARE), (II) lymphatic vessel endothelial receptor 1 (LYVE-1), and (III) toll-like receptors TLRs [11].

### 4.1. CD44

Recognized for its ability to bind to HA, cluster-determinant 44 (CD44) is widely distributed in transmembrane glycoproteins [1,2,11,54]. It is expressed in many isoforms, spread in almost all types of human cells [1,14,26]. It is these isoforms and the type of cell in which they are expressed that define the binding properties of this hyaladherin [55]. The CD44 intracellular domain interacts with the cytoskeleton, so when its extracellular domain binds to the ECM HA, a link is created between the cytoskeletal structures and the biopolymer [1].

The affinity of HA to CD44 depends on its MW; the increased avidity of the HA-CD44 bond is related to the increase in the polysaccharide chain [2,24]. Smaller fragments are also able to interact with CD44, but the effects induced on target cells are different from those caused by HMWHA [2,11]. The current hypothesis for this phenomenon is that HMWHA is able to group receptors on the surface of the cell membrane to modulate receptor activity [2,24], while small fragments of HA bind to a limited number of CD44 sites. With this action, the HA with a greater MW suppresses pro-inflammatory cytokines, and suppresses the synthesis of matrix metalloproteins (MMP), proteoglycans and prostaglandin E_2_ (PGE_2_) [24]. It has also been proven that the HMWHA-CD44 bond coats the cell membrane, forming a protective layer of HA on the cell surface that is capable of masking cell death receptors and, consequently, preventing the cell from reaching apoptosis [2]. CD44-HA interactions play an important role in the development, inflammation, recruitment and activation of T cells, and the growth of metastases in tumors [8,56].

A recent study by Sapudom et al., shows that, in addition to MW, the way HA is presented (soluble or bound to the matrix) triggers distinct cellular behavior. The studies carried out by this group show that LMWHA, but not HMWHA, is able to positively regulate cell proliferation (BRO melanoma cell lines) when in a soluble form and can increase cell invasion and adhesion in its immobilized form (linked to 3D collagen matrices) in a CD44-dependent manner. These results show that HA can promote tumor growth and invasiveness through its interactions with the CD44 receptor. Furthermore, this shows that MW and the way that HA is presented regulate the whole process [57].

### 4.2. RHAMM

The receptor for HA-mediated cell motility (RHAMM, also known as CD168), exists in several isoforms, which may not only be present in the cell membrane, but also in the cytoplasm and nucleus [1,2,9,58,59]. However, it is poorly expressed in most human tissues [26]. The cell membrane is anchored to glycosylphosphatidylinositol [59,60], containing two HA-binding domains, in which the molecule binds to CD44 less firmly than in the HA-binding domain [38]. It is RHAMM that regulates cellular responses to growth factors and plays a role in cell migration, particularly for fibroblasts and smooth cells [30,60].

The interactions of HA-RHAMM play important roles in inflammation and tissue repair [1,26], triggering a variety of signalling pathways, and thus controlling cells such as macrophages and fibroblasts [1]. On the cell surface, RHAMM interacts with CD44 and modulates cell motility, wound-healing, and signal transduction [9,51,58]. It can also perform invasive functions such as CD44 and can even replace its functions [9,58]. At the intracellular level, RHAMM binds to actin filaments, podosomes, centrosomes, microtubules, and mitotic spindle, affecting crucial cellular processes in tumorigenesis [9,58].

### 4.3. ICAM-1

The intercellular adhesion molecule 1 (ICAM-1) is considered a cell surface metabolic receptor for HA [30,32] and is widely distributed in endothelial cells and leukocytes [61]. This molecule may also be responsible for the release of HA from body fluid and plasma, which is responsible for most of its turnover throughout the body [30,32]. The binding of HA to this receptor triggers a finely regulated cascade of events that feed the endocytic vesicles [32,61]. ICAM-1 can also function as a cell-adhesion molecule, and the HA-ICAM -1 bond can, therefore, regulate the inflammatory activation mediated by ICAM-1 [32,61].

### 4.4. Other Receptors

The HA-receptor for endocytosis (HARE, also known as Stabilin-2) is capable of binding not only HA, but also other GAGs, and is involved in the release of GAGs into the circulation [1,2,13] and in the nuclear factor kappa-light-chain enhancer of activated B cells (NF-кB) [13,52]. It is found on the inner surface of endothelial cells in vascular and lymphatic vessels [2,19]. HARE mediates the systemic clearance of HA from the circulatory and lymphatic systems [62,63].

The lymphatic vessel endothelial receptor 1 (LYVE-1) is an HA-binding protein expressed in the vascular lymph endothelium and macrophages, which controls the renewal of HA by mediating its absorption from the tissues into the lymph. This helps to regulate the moisture level of the tissue [1,2,13,51]. In this way, LYVE-1 forms complexes with growth factors, prostaglandins and other tissue mediators, which are involved in the regulation of lymphangiogenesis and intercellular adhesion [1]. LYVE-1 interacts with HA, and the resulting complex can be internalized and targeted in lysosomes [51].

Toll-Like receptors (TLRs) are involved in the activation of innate and adaptive immune responses and also play roles in tumor progression and inflammation [13,64], being expressed in the membrane of all mammalian cells [51]. The smaller fragments of HA can bind to the TLR2 and TLR4 receptors of monocytes, dendritic cells and lymphocytes, thus provoking a pro-inflammatory response [30,51,65]. These receptors are widely distributed in the gastrointestinal tract, where they play an important role in mediating the response to commensal and pathogenic hosts and bacteria [54]. Although the direct physical interaction between HA-TLR was not experimentally demonstrated, it is likely that the polyanionic nature of HA mimics the canonical ligands of TLR2 and TLR 4, such as lipopolysaccharide [51]. The HMWHA-TLR interaction induces cell survival; conversely, the LMWHA-HA interaction induces inflammation and cell death [51].

## 5. HA Potential as Therapeutic or Coadjutant Agent

Due to its unique biological and physicochemical properties and safety profile, HA represents a useful and emerging tool to improve drug administration, showing significant clinical viability [1,66,67]. In the field of biomedical applications, HA has become a carrier of great interest due to its advantages, such as: (I) biodegradability, (II) biocompatibility, (III) non-immunogenicity (IV) ease of chemical modification, (V) high hydrophilicity, (VI) intrinsic targeting properties due to selective interactions with its receptors, and (VII) its exclusive rheological behavior [11,26,62,68,69]. 

However, its low stability and short biological half-life limit its application. To overcome these problems, HA can be modified to improve its stability and obtain derivatives with superior properties [70]. The HA molecule contains a carboxylic acid, a primary alcohol and an amide, in each monomer, which are important for biological functions and available for chemical modifications [38,53,68,71]. Carboxylic acid and primary alcohol are important for recognition by hyaladherins, while amide improves the adhesive properties of the molecule, and is the least used for chemical modifications [38].

Considering that HA has several biological functions, drug conjugates with HA can perform their therapeutic functions in the same way [1]. The combination of active ingredients and HA aims to develop prodrugs with better physicochemical properties, better stability and increased therapeutic efficacy compared to free drugs [1]. Conjugates have a controlled release and targeted effect, enabling the delivery of various drugs to pathological sites [71]. On the other hand, the absorption of water by HA leads to an expansion of its volume, forming a viscoelastic gel, which mimics the structure of the native tissue. These gels can be used in regenerative medicine to replace tissues. In these cases, HA will act as a signalling molecule for cell proliferation and ECM remodelling [69]. However, HA has a very short blood half-life; most drug conjugates for this polymer have been developed for local rather than systemic administration [1,68]. One strategy to circumvent the degradation in the body is to add a nitroxide-containing substance or an HYAL inhibitor to HA [71].

Nanoparticles represent a promising type of drug delivery system, which can be formulated with HA. HA can form a constituent element of the nanoparticle, but it can also be used to cover the nanoparticles to improve the targeting efficiency and therapeutic action of encapsulated drugs [1]. The selective delivery of drugs through nanoparticles formulated with HA is improved due to the interaction of HA with hyaladherins. Generally, this type of conveyor has a negative surface charge, which prevents elimination by the reticuloendothelial system (RES) [72].

As shown previously, HA is a molecule with a wide range of properties that has several applications in the field of biomedicine. These properties include cell interaction, such as cell differentiation and proliferation, and their biological activities, including their ability to lubricate, hydrate, and interact with various receptors present on the cell surface. It is the interaction with these receptors that facilitates the targeted delivery of drugs, facilitating their internalization in target locations. In this article, the strategies used in the area of inflammatory pathologies, in which HA plays an important role, will be explored.

### 5.1. Inflammatory Joint Diseases

HA is one of the main lubricating agents of ECM in synovial fluid [1,26]. The functions of HA in the joints include lubrication, which serves as a space filler to allow the joint to remain open and regulate cellular activities [23], as its viscoelasticity absorbs mechanical impacts and avoids friction between bone ends [1,26,73,74]. During the inflammatory processes, uncontrolled turnover due to decreased pH and increased ROS can lead to losses in the thickness and viscosity of the HA barrier [75], leading to disorders such as osteoarthritis (OA) or rheumatoid arthritis (RA).

The safety, tolerability, and efficacy of HA-based formulations for the treatment of various types of joint diseases have been validated in several studies. The mechanism involved in HA’s effectiveness in the treatment of this type of joint pathologies is due to its capacity for chondroprotection [41].

#### 5.1.1. Osteoarthritis

Osteoarthritis (OA) is an inflammatory pathology of the diarthrodial joints, characterized by chronic and progressive cartilage degeneration, the formation of osteophytes, subchondral sclerosis, bone hypertrophy and changes in the synovial membrane [11,76,77]. HA is found in the joint, providing viscoelastic properties to the synovial fluid [23,24,78]. The onset of OA, associated with reduced HA synthesis and increased degradation, leads to a shift in distribution towards a lower average MW in the synovium, synovial cavity and cartilage, which consequently decreases the mechanical and viscoelastic properties of the synovial fluid in the affected joint [23,24]. The permeability of the synovial membrane increases in response to inflammation or injury, which leads to increases in the infiltration of plasma fluids and, in turn, decreases the concentration of HA in the joints. This decrease in HA concentration and/or MW can result in changes in the synovial fluid viscoelasticity, leading to joint dysfunction [16].

Damaged cartilage has a reparative capacity; however, it is a limited capacity, largely because it has a low cellular concentration and little vasculature [22,79]. Injections of exogenous HA have been used clinically to relieve the macerated functions of depolymerized, endogenous HA [23,76]. In fact, exogenous HA does not restore or replace all the properties and activities of the endogenous molecule of synovial fluid, but it can produce satisfactory pain relief through several mechanisms. These mechanisms include anti-inflammatory effects and the maintenance of viscoelasticity. However, there is a clear heterogeneity in the therapeutic trajectory for patients with OA after HA injections. Some studies report a general beneficial effect, while others report only a small benefit. One of the main reasons for this variable effect lies in the HYAL levels present in the patient’s synovial fluid [23]. Thus, due to this variability, combinatory strategies with drugs are being pursued.

Liposomes loaded with celecoxib (Clx), incorporated in an HA gel for intra-articular administration (IA), have been developed by Dong et al. The obtained results show that the incorporation in the HA gel delayed the release of Clx. In vivo studies, using rabbits with knee OA as a model, showed that the combination of liposomes with Clx and HA was more effective in controlling pain and protecting cartilage when compared with liposomes loaded with Clx or HA alone [80].

Another study reports that PEGylated self-assembled kartogenin micelles (PEG/KGN) are capable of inducing chondrogenesis in human stem-mesenchymal cells. Based on this information, Kang et al., developed an HA hydrogel containing PEGylated micelles of kartogenin (HA/PEG/KGN). In vitro, HA/PEG/KGN were also found to be enzymatically degraded by the action of collagenase and hyaluronidase. In vivo, after the intra-articular injection of HA/PEG/KGN in the joint, it was found that OA progression was suppressed in rats with surgically induced OA when compared to HA hydrogel injection in the absence of PEG/KGN. In fact, it is known that KGN has the ability to induce chondrogenesis in human stem-mesenchymal cells by binding to filamin A, and that HA-CD44 interactions promote the recruitment of filamin A. Thus, these favourable interactions can lead to the intracellular delivery of KGN [81].

Kim et al., studied the effects of an HMWHA system containing sulfasalazine (SASP/HA) in synoviocytes inflamed by lipopolysaccharide (LPS) and in a mouse model with monosodium iodoacetate (MIA)-induced OA. In vitro studies show that SASP/HA was able to inhibit proinflammatory cytokine levels (including metalloproteinase-3 (MMP-3), cyclooxygenase-2 (COX-2), interleukin-6 (IL-6) and tumor necrosis factor α (TNF-α)). In vivo, after the intra-articular injection of SASP/HA, there was relief in the inflammatory responses, which prevented the destruction of the cartilage and progression of the disease [82].

In cartilaginous tissue, in addition to HA, collagen has important mechanical properties. For this reason, Mohammadi’s group developed scaffolds of HA and collagen where prednisolone (PD) and transforming growth factor β3 (TGF-β3) were incorporated for the regeneration of cartilage in rats in two different pharmaceutical forms: implantable discs and injectable thermosensitive gels. Implantable discs were considered more effective than thermo-sensitive gels due to their durability at the inflammation site, better mechanical properties, and similarity to cartilage. In vitro, it was shown that a sustained release of TGF-β3 was achieved for more than 96h. In vivo, implantable discs, with TGF-β3 and prednisolone, were most effective. Radiographic images and pathological samples, after four months, show that all formulations have a therapeutic effect. However, the best result (osteoarthritis score and, global score) was observed in the samples treated with TGF- β3 [83].

Based on the conjugation of HA with methotrexate (MTX), using 4,7,10-trioxa-1,13-tridecanediamine (PEG13) as a linker and Asn-Phe-Phe as a peptide (a peptide that is essential for lysosomal enzymes, to ensure that the bond between HA and MTX is broken), Homma et al., found that intra-articular injection of HA-MTX was able to reduce knee-swelling in a mouse model with antigen-induced arthritis (AIA). On the contrary, free MTX, HA or a mixture of HA and MTX, had very few or no effects, probably due to the rapid removal of the joint cavity [84]. Later, the HA-MTX conjugate was optimized for the DK226 conjugate (using NH(CH_2_)_2_NH as a linker and α-Phe-Phe as a peptide). In vitro, it was shown that the proliferation of synoviocytes is similar to human fibroblasts (human fibroblast-like synoviocytes (HFLS)), and synovial sarcoma cells (SW982) were inhibited in the presence of DK226. It was also found that MTX was released by incubation with rabbit synovial tissue homogenate or synovial fluid at pH 4.0. In addition, in both AIA and collagen-induced arthritis (CIA), the intra-articular injection of DK226 was able to reduce swelling and inflammation, which was not the case with MTX or the mixture of MTX and HA [85].

Using an experimental model of OA, in vivo, Siracusa et al., studied the protective effect of a carnosine (CARN) and HA (FidHycarn) conjugate on inflammatory response and cartilage degradation. The oral treatment of rats with knee-induced OA through an MIA solution improved the macroscopic signs, thermal hyperalgesia, and weight distribution of the hind paw, and decreased histological and radiographic alterations. The results show that the levels of cytokines and chemokines, as well as oxidative damage, were reduced in the presence of FidHycarn when compared to the administration of CARN, HA, or the mixture of CARN and HA. In this particular case, HA has the ability to protect CARN against enzymatic degradation (serum carnosinases), increasing the effectiveness of FidHycarn [86].

The cytokine response modifier A (CrmA) is a serpin-like protease inhibitor encoded by the bovine smallpox virus, which can bind to the interleukin-1 β (IL-1β) conversion enzyme and, as a consequence, inhibit the generation of IL-1β; therefore, CrmA can have beneficial effects in the treatment of OA. Zhou and his team developed HA and chitosan (CS) nanoparticles, which incorporated plasmid DNA encoding CrmA (HA/CS-CrmA). In vitro studies show that HA/CS-CrmA safely transfect synoviocytes and release plasmid DNA in a controlled manner over 3 weeks. In a rat OA model constructed of the previous anterior cruciate ligament transection (ACLT) in vivo, HA/CS-CrmA is also capable of significantly inhibiting cartilage damage, synovial inflammation, and loss of type II collagen. It was also found that in the presence of HA/CS-CrmA, the levels of IL-1β, MMP-3 and metalloproteinase-13 (MMP-13) in the synovial tissue were regulated [87].

Due to inflammation, there is a decrease in pH of the synovial fluid, a reason that led Zerrillo et al., designing poly(lactic-co-glycolic acid) (PLGA) nanoparticles, where they incorporated ammonium bicarbonate (NH_4_HCO_3_) and HA. The porous surface of the PLGA nanoparticles allows for the entry of water and H_3_O^+^ molecules. The reaction between H_3_O^+^ and NH_4_HCO_3_ leads to a neutralization of the pH value and to the breakdown of the nanoparticle structure, allowing for the exit of HA. The results showed that the developed NPs had no toxicity (both in vitro and in vivo). The nanoparticles, with and without HA, led to a reduction in disease progression; however, the presence of HA had more evident effects [88].

A hydrogel of HA and particles of PLGA and oleic acid was developed by Mota et al., for viscosupplementation in OA, using intra-articular administration. The studies carried out in vitro showed a sustained profile of HA release and reduced risk of hemolysis. Anti-inflammatory studies in vivo (cotton-pellet-induced granuloma method) showed a greater inhibition of PLGA particles with HA when compared to an HA solution. The mechanism of action has not been studied; however, it is believed to be mediated via the CD44 receptor [89].

In vivo, Tolba et al., studied the effects of HMWHA on the osteoarthritic changes induced in the temporomandibular joint (temporomandibular joint (TMJ)) with an intra-articular injection of complete Freund’s adjuvant (CFA). Male rats were used, divided into three groups (control, OA in TMJ and treatment group). In the treatment group, three intra-articular injections of HMWHA were used, administered weekly. In the group in which OA was induced without treatment, there was abnormal disorganization and stratification in the tissue layers, and bone sclerosis. In the group in which HMWHA was administered, there was a reduction in pathological changes and restoration of the normal structure of TMJ, with a simultaneous decrease in MMP-3 levels [90]. Thus, the obtained results show the chondroprotective effects exerted by HA.

Faust et al., used a peptide-polymer cartilage-coating platform to locate HA on the cartilage surface. The aim of the study was to increase the effectiveness of the peptide-polymer platform in reducing OA progression in a post-traumatic mouse model of OA. The peptide-polymer is composed of an HA-binding peptide (HABP) conjugated to a heterobifunctional polyethylene glycol (PEG) chain and a collagen-binding peptide (COLBP). After creating a library of different peptide-polymer conjugates, the one that showed more affinity for HA was HABP2-8-arm PEG-COLBP. After labelling with biotin, HABP2-8-arm PEG-COLBP was found in both cartilage defects and synovium, 24 h after intra-articular injection. In vivo treatment with HABP2-8-arm PEG-COLBP and the clinical HA comparator Orthovisc reduced levels of IL-6, IL-1β and MMP-13, and increased levels of aggrecan, compared with animals treated with solution saline. Cartilage degeneration was also reduced by HABP2-8-arm PEG-COLBP and Orthovisc. However, in elderly mice, the therapeutic effectiveness of HABP2-8-arm PEG-COLBP was similar to its effectiveness in young mice, but Orthovisc was less effective. These data support the theory that HABP2-8-arm PEG-COLBP is effective in reducing the progression of OA [91].

Epigallocatechin-3-gallate (EGCG) is a polyphenol capable of eliminating ROS and preventing the oxidative damage induced by inflammation; it was previously shown to suppress inflammation in various types of cells. Using this information, Jin et al., investigated EGCG-loaded HA/gelatine (HTG-E) hybrid hydrogel when HA works as a lubricating component for cartilage. In vitro, the developed hydrogel protected chondrocytes against IL-1β. In vivo (surgically induced OA model), the intra-articular injection of the hydrogel was able to induce chondrogenic regeneration and minimized cartilage loss, probably due to the hydrogel’s ability to absorb large amounts of water, which may have contributed to the sequestration of inflammatory cytokines. Furthermore, due to the adhesive properties of the hydrogel, its retention time in the joint is increased, which makes the effects of EGCG more evident [92].

To improve the lubrication properties of HA to treat OA, Zheng et al., grafted 2-methacryloyloxyethyl phosphorylcholine (MPC) onto the HA with two different MWs (HAMPC). The lubrication assessment shows that, compared to HA, the friction coefficient of HAMPC was reduced. In vitro, it was also found that HAMPC was biocompatible and can positively regulate the anabolic genes of the cartilage (aggrecan and collagen type II alpha 1 chain (COL2A1)) and deregulate catabolic proteases (MMP13 and a desintegrin and metalloproteinase with thrombospondin motifs 5 (ADAMTS5)) of the cartilage and pain-related genes. The low MW HAMPC (lHAMPC) showed a greater capacity to regulate pain-related genes; however, on the other hand, the high MW HAMPC (hHAMPC) has a greater lubrication capacity and better anti-inflammatory activity [93].

Based on the lubrication properties of HA, Yang et al., developed a novel drug delivery microcarrier with pathological-state-responsive switches. In this system, the temperature-responsive hydrogel (poly(N-isopropylacrylamide) (pNIPAM)) was used to form an inverse opal-structure microsphere scaffold to increase the drug-loading efficiency, while HA (methacrylate anhydride-modified HA) was employed as a vehicle to encapsulate diclofenac sodium. In vitro, the DS particles were placed at 31 °C (average joint temperature) for 20 min, at 37 °C (physiological temperature after exercise) or 39 °C (temperature after exercise or OA) for 5 min to form a cycle detection temperature. The DS release rate was faster in pNIPAM that at room temperature, since pNIPAM can shrink when the temperature is above the volume phase transition temperature (VPTT), and thus the DS can be released. In addition, the amount of released DS decreased with increasing HA concentration. Chondrocyte studies show that pNIPAM, pNIPAM+HA, pNIPAM+HA&DS have sufficient biocompatibility, with no evident differences between the different groups, and that particles loaded with DS did not affect the proliferation of chondrocytes. In vivo (Spregue-Dawley rats, intra-articular injection, 6 weeks of treatment), the pNIPAM+HA and pNIPAM+HA&DS groups showed a positive morphological change, and the pNIPAM+HA&DS group showed the best effects, characterized by less damage and erosion, more cells, and a smoother surface. The results obtained with Safranin O-Fast Green staining showed that the groups pNIPAM+HA and pNIPAM+HA&DS showed an increase in GAG deposition and the attenuation of cartilage matrix degradation. Treatment with pNIPAM+HA or pNIPAM+HA&DS significantly increased the expression of aggrecan and collagen II. After treatment, the concentrations of TNF-α, protein of the cartilage oligomeric matrix (COMP), type II collagen C-terminal telopeptides (CTX-II) and metalloproteinase-1 (MMP-1) in the groups pNIPAM+HA and pNIPAM+HA&DS decreased [94].

Xu et al., evaluated the therapeutic efficacy of platelet-rich plasma (PRP) combined with HA for the treatment of knee OA (KOA). In this study 122 patients, divided into three groups (HA group, PRP group, PRP+HA group) were treated by intra-articular injection (three injections with a 1-month interval) and assessments were made before the injection and at 6 and 12 months after injection. The results showed that PRP and PRP+HA can inhibit synovial inflammation; however, PRP+HA inhibits inflammation more effectively. In these two treatment groups, there was a reduction in the levels of IL-1β, tumor necrosis factor α, matrix metalloproteinase-3 and tissue inhibitor of metalloproteinase-1 (MMP-1), with this decrease being more evident in PRP+HA. Treatment with PRP+HA also improved local synovial hyperplasia and blood flow, with a reduction in the incidence of adverse reactions [95].

To overcome the side effects of diclofenac and dexamethasone (DEX), Chang and his collaborators developed hydrophilic/hydrophobic dexamethasone with diclofenac-encapsulated liposomal-nanoparticle-containing HA (HA-Lipo-DIC/DEX). The presence of HA in nanoparticles gives it viscoelastic properties and joint lubrification capacity. The system was shown to be able to initiate anti-inflammatory action after 4 h, maintaining a controlled release of the drug for 168 h. Chondrocyte studies have shown that HA-Lipo-DIC/DEX has no toxicity, and is capable of inducing cell replication. In vivo (mice with AO induced by CFA, intra-articular injection), HA-Lipo-DIC/DEX has been shown to be able to reduce inflammation for a period of 4 weeks after intravenous injection, compared to DEX or liposomal DEX [96].

El-Gogary et al., developed HA nanocapsules loaded with celecoxib. In vitro studies show that, with the nanocapsules, a prolonged release of celecoxib (up to 7 days) is achieved, with an increased internalization rate. In vivo (monoiodoacetare-induced osteoarthritis rat model), after intra-articular injection, celecoxib nanocapsules show a superior anti-inflammatory activity to celecoxib. It was also found that celecoxib nanocapsules remain in the affected joint, due to the interactions established between the HA and macrophages present in the joint [97].

Cai et al., studied the role of magnoflorine combined with HA-gel (HA-gel+magnoflorine) in attenuating the cartilage degeneration induced by anterior cruciate ligament transection (ACTL) in a mouse model. After treatment with intra-articular injection (Sprague Dawley rats) of HA-gel+magnoflorine, there was an inhibition of pro-inflammatory cytokines and a positive regulation of interleukin-10 (IL-10, anti-inflammatory cytokine), showing that HA-gel+magnoflorine has a protective effect on the cartilage matrix. In vitro (primary chondroprogenitor cells), there was also proliferation, chondrogenesis and migration of chondropogenitor cells, which has an indirect effect on maintaining the integrity of the subchondral bone (SCB) [98].

Injectable hydrogel was synthesized by the in situ crosslinking of N-carboxyethyl chitosan (N-chitosan), adipic acid dihydrazide (ADH), and hyaluronic acid–aldehyde (HA-ALD) by Mou et al. In vitro (chondrocytes), the obtained results showed a tendency to increase the proliferation of chondrocytes in the hydrogel, compared to the control group. In vivo (Sprague Dawley rats), after subcutaneous injection, it was found that complete degradation of the hydrogel only occurred between days 24 and 30. After the induction of AO (single monosodium iodoacetate (MIA) injection), treatment with the hydrogel was shown to inhibit inflammatory cytokines (TNF-α, IL-1β, IL-6 and IL-17) in synovial fluid and cartilage, compared to treatment with only HA. Hematoxylin and eosin (H&E) staining showed that the control group (without treatment) with an articular surface was unsmooth: some cartilages were missing and damaged, there was marked fibrosis, and the surface cells of cartilage reached necrosis. In the HA-treated group, the cartilage surface was already intact, but the cells were loose and irregular. In contrast, the hydrogel-treated group had a smooth, intact joint surface with a thick layer of cartilage and organized cells [99].

Table 1 summarizes the studies presented throughout this review for the treatment of osteoarthritis, where the beneficial role of HA is evidenced.

#### 5.1.2. Rheumatoid Arthritis

Rheumatoid arthritis (RA) is an autoimmune disease that causes bone destruction, hyperplasia of synovial membranes and damage to cartilage. Although the exact mechanism of this pathology remains unknown, abundant activated macrophages in inflamed joints are known to play a crucial role in disease progression through the production of pro-inflammatory cytokines, such as TNF-α, IL-1β and IL-6 [66,100]. Therefore, inhibiting the secretion of pro-inflammatory cytokines from activated macrophages has been the main therapy for RA. CD44 is an adhesion receptor that is often overexpressed on the surface of activated macrophages in patients with RA. Thus, due to its ability to bind to CD44, HA was selected as a target molecule for the development of new RA therapies [66].

The search for new treatments for this pathology led Zhou and his collaborators to develop a system of targeted prednisolone (PD) delivery, which consists of solid lipid nanoparticles (SLNs) coated with HA (HA-SLN/PD). Studies have shown that the developed system remains stable in circulation for long periods, and accumulates selectively in the inflamed tissue, where PD ends up being selectively released. In addition, in a mouse model with collagen-induced arthritis, it showed good efficacy, based on joint analysis, pannus formation, bone preservation and pro-inflammatory cytokine measurement. It was also demonstrated that the therapeutic effects of HA-SLN/PD are superior to those observed with SLNs/PD and PD alone, which shows the capacity of the system to reach the arthritic joints and selectively release the drugs in these places [101].

Starting from the same principle, Gouveia et al., developed pH-sensitive liposomes functionalized with HA, where prednisolone disodium phosphate (PDP) was incorporated for intravenous or intra-articular administration. In vitro studies have shown that the liposomes’ ability to respond to pH allowed for a significant increase in the release of the drug over time. Cellular studies have shown that HA-conjugated liposomes were effectively uptaken by macrophages and fibroblasts (by interaction with the CD44 receptor). The results show that this strategy improves the efficiency and increases the bioavailability of PDP in situ [102].

Based on the activated macrophages and the fact that they overexpressed the CD44 receptor, Yu et al., developed polymeric nanoparticles coated with acid-sensitive HA, using dexamethasone (HAPNPs/Dex) as active ingredient for the intravenous administration of injection. Studies carried out by this group showed that the in vitro release rate of Dex increased significantly with a decrease in pH from 7.4 to 4.5. In cell uptake studies, stronger signals were obtained from the activated macrophages treated with HAPNPs, indicating that these nanodevices could be effective in targeting activated macrophages. On the other hand, non-functionalized nanoparticles had a much lower internalization rate. This indicates that HAPNPs interact favourably with the CD44 receptor. In vivo, therapeutic efficacy studies have shown that the infiltration of prominent inflammatory cells, bone damage and cartilage damage was reduced in the ankle joints of rats with adjuvant-induced arthritis (AIA) when treated with HAPNPs/Dex [66].

It has been reported that γ-secretase inhibitors can improve RA by suppressing the Notch signaling pathway. Using this information, Heo et al., developed HA nanoparticles (HA-NPs) carrying an γ-secretase inhibitor, N-[N-(3,5-difluorophenacetyl-ι-alanyl)]-S-phenyl-glycine t-butyl ester (DAPT) (DNPs). In vivo, biodistribution studies showed that DNPs were effectively accumulated in the inflamed joint, after systemic administration to mice with collagen-induced arthritis (CIA). When comparing therapeutic efficacy, where clinical scores, tissue damage and neutrophil infiltration were evaluated, the results showed that DNPs were more effective than DAPT. It was also found that DNPs significantly reduced the production of pro-inflammatory cytokines [103].

Alam and his team developed mineralized nanoparticles (MP-HANPs), composed of PEGylated HA (P-HA) and calcium phosphate, for the administration of MTX. Calcium phosphate acts as a barrier to diffusion, which causes MP-HANPs to release MTX only under acid-neutral conditions. The in vitro results showed that MP-HANPs were internalized by receptor-mediated endocytosis, namely, the CD44, stabilin-2 and RHAMM. The developed structures allowed for a greater accumulation and more controlled release of MTX, but with the paws of CIA mice, with a simultaneous, lower accumulation in the liver, which indicates good biodistribution [104].

Fan and his team developed HA/curcumin (HA/Cur) nanomicelles. It was shown that the developed nanomicelles have good biocompatibility and promote the proliferation of chondrocytes. After the intra-articular administration of nanomicelles, there was a significant decrease in edema in rats with complete Freund’s adjuvant (CFA)-induced arthritis. The friction between the cartilage surfaces decreased, which culminated in the protection of cartilage, an effect that is essentially due to the HA action. There was also a decrease in the expression of cytokines related to vascular endothelial growth factor (VEGF), which resulted in a marked decrease in the inflammatory response, with Cur being responsible for this effect. The results show a beneficial effect of using HA and Cur in one system. In addition, in this case, the solubility of Cur is increased, thereby increasing its bioavailability [105].

A study using the FidHycarn conjugate in OA treatment previously been reported. [87] Using the same conjugate, Impellizzeri et al., evaluated its protective effect on the modulation of inflammatory response in mice subjected to collagen-induced arthritis (CIA). Treatment with FidHycarn improved clinical signs (arthritic index), behavioral deficits (locomotor abilities, pain sensitivity testing, thermal hyperalgesia) and decreased histological and radiographic changes. There was also a reduction in oxidative damage. In addition, the levels of pro-inflammatory cytokines and chemokines and COX-2 were also reduced [106].

Another modality for RA treatment is photodynamic therapy, which involves the use of a dye (photosensitizer (PS)). Visible light is used to excite the PS, which leads to the formation of ROS, which ultimately induces cell death. However, the injection of PS into the joint results in its rapid outflow from the joint, leading to a low therapeutic index. To increase its retention in inflamed tissues, Schmitt et al., developed chitosan (CS) nanogels with HA on the surface, incorporating three photosensitizers. In vitro, the selective delivery of PS to macrophages and the photodynamic destruction of these cells were observed, due to HA’s interaction with CD44 receptors (overexpressed in macrophages). In vivo (antigen-induced arthritis (AIA) mice), it was shown that the intra-articular injection of free PS resulted in its rapid clearance from the joints, while the PS encapsulated in the nanogel was retained in the inflamed joints for longer periods, which led to a reduction in inflammation [107].

As previously reported, HA is not only used in nanoparticles, as in the previously reported examples, but also by direct conjugation to active ingredients, improving their properties in circulation. Thus, Shin et al., prepared an HA-methotrexate conjugate (HA-MTX), which has the ability to be cleaved in slightly acidic environments, as in the case of RA. The studies carried out by this group have shown that the release of MTX from the conjugate is pH-dependent, obtaining higher values under acidic conditions (pH 5.0) compared to the physiological pH. In addition, it was found that the conjugate can specifically bind to CD44 in inflamed cells and that HA-MTX is selectively accumulated in the inflamed joints of CIA mice. This results in the negative regulation of inflammatory cytokine levels and decreased cartilage damage [108].

A flare-responsive hydrogel was produced by Joshi et al., using triglycerol (TG-18) and triamcinolone acetinide (TA) as an active ingredient. It was demonstrated that a single dose of hydrogel loaded with TA reduced the arthritis activity in the paw of a mouse model K/BxN of IA when compared with free TA. The cumulative release of TA was also found to be only 20% after 10 days in vitro. However, the initial release of the drug in non-inflammatory conditions, both in vitro and in vivo, was also demonstrated, which may be due to the enzymatic degradation of TG-18 [109].

Pandey et al., developed hyaluronate-functionalized hydroxyapatite nanoparticles laden with MTX and teriflunomide (TEF): HYA-HAMT-NPs. In in vitro studies, (RAW 264.7 cell line) HYA-HAMT-NPs showed less cell viability, compared to free drugs, which may be due to the synergistic effects between MTX and TEF. It was also found that cell viability is lower in the presence of lipopolysaccharide (LPS), which indicates that HA is targeted due to its macrophages, which express CD44. The results obtained in vivo, after intra-articular injection, showed that HYA-HAMT-NPs has the ability to prolong the retention time in the joint. Controlling the ankle diameter and arthritis index shows that HYA-HAMT-NPs is capable of reducing inflammation and swelling. It was also found that HYA-HAMT-NPs have a greater anti-inflammatory capacity compared to NPs without the HA coating, reducing the levels of TNF-α, IL-6 and IL-1β. Liver histology also showed that HYA-HAMT-NPs reduces hepatotoxicity compared to commercial oral treatments (FOLITRAX-10 and AUBAGIO) [110].

The [aminocarbonyl)amino]-5-(4-fluorophenyl)-3-thiophenecarboxamide (TPCA-1) has the ability to suppress inflammation by inhibiting the nuclear factor-к signaling pathway (NF- кB, activates osteoclasts that cause bone damage). Based on this information, the Wang et al., Group developed a combined therapy of TPCA-1, gold and HA, for which they used gold nanocages (AuNCs) to transport TPCA-1 (T). AuNCs loaded with TPCA-1 were modified with HA and peptides (P)–HA-AuNCs/T/P. The anti-inflammatory capacity of the developed system was assessed in an adjuvant-induced arthritis (AIA) mice model (intravenous injection). Distribution studies have shown that HA-AuNCs/T/P accumulate in the inflamed paws, suppressing joint swelling and relieving cartilage and bone damage. The use of HA is intended to increase the targeting of inflamed macrophages that express the CD44 receptor. However, liver cells also express these receptors, so HA-AuNCs/T/P also accumulated in the liver [111].

The previously presented studies for RA treatment, with the beneficial evidence of HA, are summarized in Table 2.

#### 5.1.3. Other Disorders

Biosomes are flexible and deformable vesicles composed of phospholipids and bile salts that structurally resemble liposomes. They have been extensively studied to improve the bioavailability of various therapeutic agents, since they offer an alternative to supplying drugs to tissues with low permeability, such as the joint capsule. Yang et al., developed biosomes consisting of 1,2-stearoyl-3-trimethylammonium-propane (DOTAP) and coated with HA for the targeted delivery of tripterin (Tri) to the inflamed joint: HA@Tri-BLs. In vitro, HA@Tri-BLs showed a longer circulation time and an increase in the intra-arthritic bioavailability of Tri, compared to Tri-BLs. HA@Tri-BLs was also shown to have significantly higher antiarthritic efficacy than Tri-BLs, resulting in reduced inflammation in vivo after intravenous injection in rats receiving an injection of complete Freund’s adjuvant (CFA) [112].

The study by Storozhylova et al., reports the development of a fibrin and HA hydrogel formed in situ containing nanocapsules (NCs, consisting of an olive oil core surrounded by an HA shell) loaded with galectin-3 inhibitor (Gal-3i). In vivo (acute synovitis rat model induced with carrageenan), after intra-articular administration, NC Gal-3i was found to reduce inflammation after 4h compared to the control group. Swelling was also effectively inhibited by the fibrin and HA hydrogel with NC Gal-3i compared to the control group [113].

Gout is a metabolic disorder of the purine accompanied by high levels of uric acid (UA) in the blood and the precipitation of sodium monourate crystals (monosodium urate (MSU)) in the joints and tissues. In the absence of treatment, progressive deterioration of the tissues and bones of the joints can occur, causing chronic synovitis, bone erosions, damage to the cartilage and tophi formation [114,115]. The work by Wang et al., shows that N-butyrylated HA (BHA) has an anti-inflammatory effect, leading to a reduction in joint swelling and a reduction in serum levels of IL-1β, IL-8, gamma interferon (IFN-γ) and monocyte chemoattractant protein 1 (MCP-1). The intraperitoneal injection of BHA suppressed UA production, reducing the activity of xanthine oxidase (XO, which catalyses UA production). BHA has been shown to exhibit anti-inflammatory, antioxidant and antihyperuricemic effects in vivo [115].

### 5.2. Tendinopathy

Tendons are “cords” of connective tissue that connect the muscle to the bone and can withstand stressful situations. During muscle contraction, it is these structures that assist in the movement of bones and joints [116]. The term tendinopathy describes a clinical condition characterized by pain, swelling and functional limitations of the tendon and nearby anatomical structures [117,118]. HA is actively secreted by the tendon sheath and, similarly to what happens in the joints, as an important component of synovial fluid, it allows for smooth sliding and provides nourishment to the tendon [119]. Although the literature is still scarce, there are reports that LMWHA is an effective tool in the treatment of various tendinopathies [117].

The efficacy and safety of peritendinous HA injections (500–730 kDa) in reducing pain in patients affected by lateral elbow, Achille or patellar tendinopathy, was evaluated in a study conducted by Fogli et al. In this study, patients were treated with a cycle of ultrasound-guided peritendinous injections (one injection per week for three consecutive weeks). The results show a significant reduction in the visual analogue scale (VAS) and sagittal thickness in all types of tendinopathies under study. Neovascularization decreased for each tendon at 14 and 56 days, except for the patellar tendon. This shows that peritendinous injections provide significant pain relief and reduced tendon thickness and neovascularization, in a safe and well-tolerated manner [120].

After surgery on the tendons of the hand, there may be a limitation of hand function caused by postoperative tendon adhesion, which is mainly attributed to tendons that adhere to the surrounding tissues during the healing process, limiting joint mobility. One approach to the prevention of postoperative tendon adhesion is the use of multifunctional nanofiber membranes (NFM), which allow for inhibition of the binding and penetration of fibroblasts. In this context, Chen et al., developed HA nanofiber membranes loaded with different percentages of ibuprofen (20%-HAI20FB, 30%-HAI30FB and, 40%-HAI40FB). The studies conducted by this group show that, except for HAI40FB, all the developed NFMs showed excellent effects in preventing fibroblast binding and penetration, preserving high biocompatibility without influencing cell proliferation. Although HAI40FB shows an improvement in mechanical properties over other NFMs, it did exhibit cytotoxicity. In a rupture model of the flexor tendon of the rabbit HAI30FB, it was shown to function as a physical barrier membrane, simultaneously inhibiting inflammation and allowing adhesion formation, compared with a commercial membrane (Seprafilm^®^) and NFMs without ibuprofen [121].

### 5.3. Intervertebal Disc injury

The spine, the body’s main support, is composed of individual bone segments (vertebrae), ligaments and discs. The intervertebral discs are located between the vertebrae. Each of these discs is composed of three main components, including the nucleus pulposus (NP) [122,123], with a gel-like texture, which give the column flexibility and mechanical strength [123]. Degeneration of the intervertebral disc (IVD) is mediated by inflammation, which modulates glycosylation and induces hyperinnervation and sensory sensitization, along with the production of neutrophins, which results in discogenic pain [124,125].

Using a mouse model with an intervertebral disc injury, Mohd and his team evaluated the effectiveness of an HA hydrogel for pain relief. The results demonstrated that the hydrogel could reduce nociceptive behavior, alter glycosylation and the main pathways for inflammatory signalling, and lead to the attenuation of inflammation and regulation of matrix components [124].

Another study of NPs, by Isa et al., hypothesised that cross-linked HMWHA hydrogels can modulate the inflammatory receptor of IL-1R1, myeloid differentiation primary response 88 (MyD88) and the expression of nerve growth factor (NGF) and brain-derived neurotrophic factor (BDNF) in an in vitro inflammation model (NP cell culture, which induced inflammation with IL-1β). Studies show that the developed hydrogels do not show cytotoxicity after 7 days in culture. In addition, IL-1R1 and MyD88 were significantly suppressed and NGF and BDNF were down-regulated. The possible protection mechanism of HA is evidenced by the high expression of the CD44 receptor of NP cells after treatment with the hydrogel, which suggests that HA binding to the CD44 receptor prevents NP cells from being inflamed [125].

It has been reported that the interferon α (INFα) signalling pathway is involved in the inflammatory cascade in degenerated annulus fibrosus. The study by Kazezian et al., consists of an evaluation of the anti-inflammatory effect of HMWHA on the INFα signalling pathway in an in vivo rat-tail disc injury model. It can also evaluate the regenerative capacity of HMWHA on the IVD extracellular matrix and the glycosylation profile. The results showed that HMWHA plays an important role as an anti-inflammatory, deregulating INFα. In addition, it downregulated the expression of the pro-apoptotic, insulin-like, growth-factor-binding protein 3 (IGFP3) and the apoptosis marker caspase 3, and modulated aggrecan and hyaluronic acid link protein (HAPLN1). This led to the synthesis of ECM in the injured discs, which were treated with HMWHA [126].

### 5.4. Inflammatory Skin Diseases

In the skin, HA retains and evenly distributes water, thus preserving the volume of the skin and its elastic and flexible properties [28]. It also plays a protective role as an inhibitor of free radicals, generated upon exposure to solar radiation [26,28,74]. It has been reported that about one third of the total amount of HA (5 g) is both the dermis (±0.5 mg/g wet tissue) and the epidermis (±0.1 mg/g wet tissue) [16,37]. In the epidermis, this is metabolized and actively participates in many regulatory processes, such as cell proliferation, migration, and differentiation. In the dermis, it fills the extracellular spaces [68].

Exposure of the skin to ethanol is a common occurrence in health facilities. In addition to its use in cosmetics, ethanol is used as a vehicle in tests of contact allergies and to increase the skin’s permeability to medicines. Neuman et al., evaluated the physiological levels of damage caused by ethanol in vitro in skin cells (human A431 epidermoid skin cells) and mouse fibroblasts, and the possible repair by HA. The results report that the treatment of cells with ethanol increased cytotoxicity, as well as the release of pro-inflammatory cytokines. On the other hand, HA reduced the amount of pro-inflammatory cytokines released [127].

The work conducted by Pleguezuelos-Villa et al., aimed to develop mangiferin (natural xanthone) nanoemulsion hyaluronate gels for inflammatory disorders in the absence or presence of transcutol-P (a solubilizer associated with skin-penetration enhancement in topical dosage forms). The results show that the release of mangiferin depends on the MW of the polymer; in addition, the permeability tests (swine epidermis) showed that nanoemulsions with a low molecular weight HA improve permeation, with this effect being more evident in nanoformulations with transcutol- P. In mice inflamed with 12-O-tetradecanoylphorbol-13-acetate (TPA), the topical application of nanoemulsions attenuated of edema and leukocyte infiltration. In summary, the results show that the topical application of these formulations has an appropriate anti-inflammatory effect [128].

#### Atopic Dermatitis

Atopic dermatitis (AD) is a chronic inflammatory skin disease, characterized by intense itching, rashes and inflammatory reactions [129,130,131]. To date, there is no total therapy for the treatment of AD, due to its complex pathogenic interaction between genes that are susceptible to the patient, abnormalities in the skin barrier and immune dysregulation. Several pharmacological and non-pharmacological approaches have been used. Among the various pharmacological interventions, topical corticosteroids (CTs) are the main choice; however, they present a series of local and systemic adverse effects. As an alternative, topical calcineurin inhibitors (TCIs) appear, and can be used for longer periods in an equally effective and safe way [130,131].

The purpose of the work by Zhuo et al., was to provide tacrolimus (TCS) to the deepest layers of the skin for AD treatment. For this purpose, HA-coated nanoparticles (HA-TCS-CS-NPs) were developed. In vitro results show that HA-TCS-CS-NPs follow a sustained release when compared to TCS-CS-NPs. In addition, HA-TCS-CS-NPs show a more effective dermal targeting capacity and increase the TCS that is retained in the epidermis and dermis, showing that the presence of HA improves the therapeutic efficacy of the developed system, probably due to its mucoadhesive properties. It has also been shown that HA-TCS-CS-NPs has pronounced anti-AD efficacy, since it leads to a reduction in transepidermal water loss (TEWL), erythema intensity and dermatitis index [131].

### 5.5. Wound Healing

Wound healing is a dynamic and complex process that results in the restoration of tissue integrity and function. It is divided into three phases: (I) hemostasis and inflammation, (II) the proliferative phase, with tissue production and (III) maturation and remodelling, when the tissue is replaced by mature collagen [132,133,134]. These phases constitute a highly organized sequence of events involving many types of cells, including blood cells, epithelial and connective tissue cells, inflammatory cells, and many soluble factors, such as clotting factors, growth factors and cytokines. This process starts right after tissue damage, and lasts until the wound is completely closed and tissue regeneration is as functional as possible [132]. Sometimes, this restoration process fails and compromises the integrity of the skin, leading to potentially serious complications, such as chronic wounds [133].

During the inflammatory phase of healing, HA accumulates in the wound bed and acts as an inflammation regulator [132]. HA usually occurs in low concentrations in the bloodstream; however HA levels rise rapidly at the wound site [19]. At this stage, the main functions of HA are to modulate the migration of inflammatory cells and fibroblasts, the synthesis of pro-inflammatory cytokines and the phagocytosis of invading microbes [132,135].

The literature already presents some studies that report that HA accelerates wound healing. In this sense, Iacopetti’s group explored the effectiveness of different topical treatments on wounds experimentally created through surgery on sheep. The study consisted of the topical daily application of commercially available HA (Connettivina^®^, Fidia 2 mg/g), Manuka honey (MH: Medihoney^®^ Wound gel), Acemannan gel (AG: commercial medicine extracted from the Aloe Vera plant, Carra vet^®^ Acemanna Wound gel) or placebo (phosphate saline buffer). The results show that the topical application of HA promoted a physiological progression of the healing process in all phases of the process. Meanwhile, treatment with MH led to slightly dry wounds and promoted cell proliferation and neovascularization, with a general pro-inflammatory effect. The treatment with AG dehydrated the wounds and stimulated the late proliferation of tissues and cells; a mild pro-inflammatory effect and late neovascularization were also produced. These results demonstrate that HA improved the treatment of wounds, reducing the healing time [133].

N-butyrylated LMWHA (BHA) was used by the Gao group to assess in vitro and in vivo wound healing. BHA has been shown to significantly promote healing when compared to a commercial product (CHITIN^®^). In vitro, it appears that the production of pro-inflammatory cytokines (TNF-α, IL-1β and IL-6), associated with M1 macrophages, is reduced in the presence of BHA. This reduction indicates that M1 macrophages are transformed into M2 macrophages, which is a fundamental part of the healing process. This transformation leads to the increased expression of growth factors (e.g., transforming growth factor-β (TGF-β) and VEGF). In vivo (male wistar rats as model), improved wound healing was observed after local treatment with BHA compared to the control group, verifying that the healing process is also more efficient with BHA than with the commercial product. Thus, the results show that, unlike LMWHA, which can trigger inflammation, BHA demonstrates anti-inflammatory activities by modulating cytokine expression, indicating BHA’s ability to prevent the wound from becoming trapped in a chronic inflammatory state [136].

Li et al., developed a hydrogel of HA-poloxamer 407 (HA-POL) and tested its therapeutic effect on the healing of skin wounds. The results show that HA-POL promoted the healing of wounds on the skin and increased the accumulation of proteins in the affected area. It was also found that HA-POL has greater air permeability than a band-aid (typical wound coverage). Treatment with HA-POL improves tissue repair, avoiding the need for treatment with growth factors. HA-POL’s ability to protect wounds from bacterial infections by *E. coli* was evaluated through studies with transwells; the results show that *E. coli* migrated from the chambers to the wells below when the chambers were coated with saline. This migration was attenuated by HA hydrogel for 2–3 days and by HA-POL gel for over 5 days [137].

To accelerate the wound-healing process, Zhao et al., developed photo-responsive supramolecular hydrogels based on HA for EGF delivery. In vitro, the photo-responsive supramolecular hydrogels showed good compatibility and irradiation with UV light, and there were no significant differences in the results. In vivo assessment of the healing process through a full-thickness skin defect model showed that the controlled release of EGF from the supramolecular hydrogel leads to superior healing efficiencies compared to granulation tissue formation, growth factor levels, and angiogenesis. Additionally, it was applied to a full-thickness excisional wound model where the growth of neoepidermis was observed to extend to the center of the wounds, resulting in a reduction in the wound area [138].

Liu et al., developed an absorbable nanofibrous hydrogel for synergistic modulation of the inflammation microenvironment to accelerate the chronic healing of diabetic wounds. In vitro, the FHHA-S/Fe nanofibrous hydrogel exhibited intrinsic dual modulation mechanisms of inflammation, including antioxidant properties and the ability to transform the macrophage phenotype. In the wound bed, electrospun thioether grafted hyaluronic acid (FHHA-S/Fe) can form a nanofiber fibrous hydrogel, which is gradually absorbed over 3 days. In vivo, using a chronic diabetic wound model, the mean area of the wound after treatment with FHHA-S/Fe was much smaller than that of FHHA/Fe without grafted thioethers and the control group, especially in the early stage of healing. These results prove that FHHA-S/Fe results in an accelerated healing process, providing a simple and synergistic strategy for the effective and safe healing of chronic wounds [139].

Table 3 lists the studies on wound-healing, with HA playing an important role, that were reported in this review.

### 5.6. Inflammatory Bowel Diseases

Inflammatory bowel disease (IBD) mainly consists of Crohn’s disease (CD), and ulcerative colitis (UC) is a chronic disease of the gastrointestinal tract (GIT) [140,141,142], caused by dysfunctional or abnormal epithelial and immune responses to intestinal microorganisms [140]. UC is a condition in which the inflammatory response is confined to the colon. Inflammation is mainly limited to the mucosa, with ulceration, edema and hemorrhage along the colon. Unlike UC, CD can involve any part of the gastrointestinal tract, from the oropharynx to the perianal area [143,144]. Here, inflammation can be transmural, often extending to the serous, resulting in sinus pathways or fistula formation [144]. It is also characterized by a defect in the integrity of the epithelial barrier, resulting in the translocation of microbial and other antigens, acting as external agents [142]. Endothelial cells are known to express high levels of CD44 in inflamed IBD sites, which appear to be essential for immune cells to infiltrate inflamed tissues [140]. Therefore, HA could be an ideal carrier for the targeted delivery of drugs to the inflamed mucosa [141].

Mesalamine (also known as 5-aminosalicylic acid (5-ASA)) is a first-line drug for patients with IBD, particularly those with mild to moderate UC [140,142]. The study by Chiu et al., investigated the efficacy of its adherence, promoting healing and reducing inflammation both ex vivo and in vivo in rats with UC after combined treatment with HA and 5-ASA (IBD98-M). Treatment with IBD98-M has been shown to significantly reduce intestinal inflammation and promote colon-mucosa-healing in UC induced by 2,4,6-trinitrobenzenesulfonic acid (TNBS). In fact, it is known that the viscoelastic properties of HA can protect mucosal cells, and other anatomical structures, against chemical wounds. Treatment with IBD98-M was found to have a synergistic effect on mucosal wound-healing compared to treatment with HA or 5-ASA alone [140].

Vafaei et al., used amphiphilic HA conjugates with the ability to form self-assembled nanoparticles (NPs) in aqueous solutions (HANPs) in which budesonide (BDS) has been incorporated. In vitro, BDS-loaded HANPs demonstrated a greater anti-inflammatory effect on the secretion of IL-8 and TNF-α in inflamed cells when compared to the free drug. Increased cell uptake via CD44-receptor-mediated endocytosis was also found in inflamed CACO-2 cells when compared to CACO-2 and NIH3T3 cells [141].

In the particular case of UC, Xiao et al., developed polymeric nanoparticles that were functionalized with HA, which carried a naturally occurring tripeptide, lysine-proline-valine (KPV), which attenuates the inflammatory responses of colon cells. The obtained nanoparticles (HA-KPV-NPs) successfully mediated the targeted delivery of KPV in the colon epithelial cells and macrophages, showing that they are biocompatible and non-toxic. HA-KPV-NPs have also been shown to have combined effects against UC, accelerating mucosal healing and relieving inflammation. The oral administration of HA-KPV-NPs, encapsulated in a hydrogel based on CS and alginate, exhibited a much stronger ability to prevent mucosal damage and negatively regulate TNF-α, thus showing much better therapeutic efficacy against UC in a mouse model when compared to a KPV-NPs/hydrogel system. These results show that HA-KPV-NP/hydrogel has the ability to release HA-KPV-NP in the colon lumen, and that these nanoparticles subsequently penetrate the colitis tissues and allow the KPV to be internalized in the target cells, acting against UC [145].

Sammarco et al., demonstrated that intestinal mucosa healing can be accelerated by HMWHA. In the study carried out by this team, after verifying that HA did not present cytotoxicity, it was found that the local application of HA (via enema, in C57BL/6N or BALB/c mice) accelerated the recovery of clinical parameters and improved tissue regeneration, with reduced bleeding and hyperemia, decreased ulcers, granularity and erosions, compared to the group that received saline solution as treatment. They also demonstrated that, in response to mucosal damage, the levels of HAS-2 mRNA were dramatically increased in the affected area, which probably led to the increased synthesis of HA in active disease. However, the newly synthesized HMWHA is probably not enough to prevent the inflammatory response associated with this pathology, so the local application of HMWHA represents an efficient approach [146].

Chen et al., studied the systemic administration of HMWHA for the treatment of TNBS-induced colitis. In vivo studies with TNBS- and HA-treated C3H/HeN rats showed promising results, namely a reduction in macroscopic damage (ulceration level) and clinical (appearance of diarrhea, signs of fecal blood, perfuse bleeding from anus) and histological alterations (crypt loss, erosions, number of follicle aggregates, edema, and infiltration of cells), when compared to rats treated with 50% ethanol and TNBS together with phosphate-buffered saline (PBS). In addition, there was an increase in the expression of COX-2 and PGE_2_, in a TLR4-dependent manner [147].

An amphiphilic conjugate of HA and bilirubin (HABN) was developed by Lee et al., to study targeted modulation of the intestinal barrier in colitis. The results show that HABN accumulated in the inflamed colon epithelium and restored the epithelium barriers in a model of acute colitis (murine model of dextran sulfate sodium (DSS)-induced acute colitis). HABN modulates the gut microbiota, increasing its general richness and diversity and increasing the populations of microorganisms that are essential to intestinal homeostasis, namely, *Akkermansia muciniphila* and *Clostridium XIVα*. HABN associated with pro-inflammatory macrophages was also able to regulate innate immune responses and exerted a potent therapeutic efficacy [148].

The studies that were previously presented for the treatment of IBD, with beneficial evidence of HA, are summarized in Table 4.

### 5.7. Lung Inflammation

The airway epithelium is directly exposed to the external environment and, consequently, responds to exogenous toxic substances [149,150]. In healthy lungs, a multitude of physical, humoral and cellular mechanisms synergistically neutralize pneumococcal adhesion, tissue growth and invasion, ensuring homeostasis and functional integrity [151] through the coordinated action of various types of cells [150]. HA acts as a lubricant, is involved in tissue healing and remodeling and modulates inflammatory responses. It also plays a role in the regulation of vascular tone and mucous gland secretion [152].

Vitamin C is a physiological antioxidant of low molecular weight, which can regulate the innate immune system in the lung, as well as maintain the host defence and the function of the epithelial barrier of the airways. Sodium ascorbyl phosphate (SAP) is a derivative of vitamin C, characterized by a better stability. Fallacara and his group developed urea-crosslinked hyaluronic acid component and SAP (HA-CL-SAP) for pulmonary applications. Using Calu-3 lung-carcinoma-derived epithelia cells, it was found that HA-CL-SAP significantly decreased IL-6 and ROS levels, and improved cell-healing, in relation to HA, SAP and HA-SAP, but at a lower rate than HA-CL. The results show that HA-CL-SAP may be adequate to reduce inflammation and oxidative stress in lung diseases, such as acute respiratory distress syndrome, asthma, emphysema and chronic obstructive pulmonary diseases, where inflammation is prominent [149].

### 5.8. Inflammatory Diseases of the Oral Cavity

The oral cavity is defined as the space between the lips until the end of the hard palate. It contains the teeth, the buccal and gingival mucosa, the mandible and the hard palate, as well as the floor of the mouth and the tongue anterior to the circumvented papilla [153]. It is one of the most complex microenvironments in the human body, where interactions between the host and microbes define health and disease. Teeth are the only functional hard tissues that extend from the inside of the human body, crossing a series of other hard (bones) and soft tissues (connective tissues and epithelia), and surrounded by a rigid biofilm formed by the richest collection of bacteria outside the colon. Here, several zones are created, which work together during the inflammatory responses of the oral cavity [154]. In the oral cavity, HA supports the structural integrity and homeostasis of tissues that regulate osmotic pressure and tissue lubrication [155].

The most common forms of inflammation in the oral cavity are gingivitis (inflammation of the gums) and periodontitis (inflammation of the tissues that support the teeth) [156]. Inflammatory gingivitis generated by plaque is very common and usually recurrent, as it is mistakenly considered trivial. The cyclic recurrence of gingivitis episodes causes irreversible damage to periodontal structures [157]. On the other hand, periodontitis is characterized by inflammation and an immune response, which generally progresses to the destruction of the periodontium. It is initiated by diverse and complex biofilms that form in the teeth. The substances that are released from this biofilm gain access to the gingival tissue and initiate an inflammatory and immune response, which results in tissue destruction and bone resorption [158]. The literature reports that in patients with chronic periodontitis, a rapid loss of HMWHA occurs due to the enzymatic digestive processes [159].

HA acts as a barrier to plaque bacteria and fulfils a variety of extracellular functions that are vital for maintaining healthy gum tissue. Gengigel^®^ is a 0.2% HA-based gel used to treat gingivitis. Jain Y. evaluated the therapeutic efficacy of Gengigel^®^ in scaling. The study was carried out on 50 patients diagnosed with plaque-induced gingivitis, divided into two groups (group 1 treated with Gengigel^®^; Group 2 as a control). The results obtained over 4 weeks show a significant improvement in the evaluated parameters (plaque index, gingival index and capillary bleeding), with more advantageous results for Gengigel^®^, showing that HA is effective in the treatment of gingivitis [157].

One of the studies carried out in the area of periodontitis aimed to study the effect of the HA of various molecular weights. To do this, Chen et al., used an HA of 30, 300 and 1300 kDa in the inflammation of *P. gingivalis*-induced and in wound-healing in human gingival fibroblasts (HGFs). The results indicate that HA of 1300 kDa inhibited the production of IL-1β, IL-6, IL-8, IL-4, and IL-10, while HA of 30 and 300 kDa had no effect. The higher MW HA also inhibited the expression of NF-кB, the degradation of nuclear factor of kappa light polypeptide gene enhancer in the B-cells inhibitor, alpha (IкBα) and the activation of extracellular signal-regulated kinase (ERK) and p38 mitogen-activated protein kinases (p38 MAKP) induced by *P. gingivalis*. This shows that HMWHA can have beneficial effects on periodontal inflammation and oral wounds [158].

The effect of 0.8% HA (GENGIGEL^®^) in patients with moderate to severe chronic periodontitis, as an adjunct to scaling and root planning, was assessed by Al-Shammari et al. The study consisted of subgingival application of 0.8% HA over one week. The evaluation was carried out after 6 and 12 weeks and showed an improvement in the studied parameters (plaque index, gingival index, papillary bleeding, periodontal probing depth and clinical attachment loss). The levels of human beta defensin-2 (hBD-2, antimicrobial peptide) were significantly higher at the test sites than at the control sites [160].

After tooth extraction, alveolar osteitis (AO) is the most common complication, resulting in acute pain and discomfort [161,162]. At the extraction site, fibrin deposition and clot formation occur, which act as a physical barrier and prevent the entry of bacteria. However, the release of tissue kinases can occur during inflammation caused by extraction, which leads to clot destruction (fibrinolysis) [163]. Partial or complete disintegration of the blood clot leads to a localized inflammatory response [161,162]. The study by Suchánek et al., aimed to evaluate the treatment of AO using a pharmacological device composed of octenidine dihydrochloride (OCD) and HA. In this strategy, the OCD disinfects the wound, making the device absorbable and acting as an analgesic. In turn, HA fixes the device to the mucosa, files the wound, keeps the device stable in the presence of saliva, enhances the healing process, and acts as an analgesic. The device was administered once a day to 58 individuals diagnosed with AO until the local pain subsided (<20 mm of the Visual Analog Scale). The results show a reduction in pain after 4 days of treatment. In 8 patients, There was prolonged scarring until after the average four administrations. In patients where treatment was not as effective or failed, risk factors such as pre-treatment with Alvogyl (which contains eugenol that disinfects and helps to control the pain but causes chronic inflammation that delays healing) have been reported, as well as tooth extraction in the presence of inflammation and smoking. However, no side effects were reported during the study. Although it does not have a prolonged stability due to the presence of saliva in the oral cavity, the device proved to be capable of disinfecting the wound, fixing itself in the mucosa, filling the wound, being completely absorbed and presenting analgesic effects [161].

Subsequently, Kapitán et al., evaluated the evolution of AO healing during treatment with the device mentioned above, and highlighted several factors that may interfere with the treatment. This assessment was performed in 23 patients who underwent extraction of the lower left third molar. Patients for whom AO manifested were treated with the HA+octenidine device. Patients without risk factors recovered after two or three applications of HA+octenidine, which led to the patient’s collapse. After that, the treatment lasted for 3 days, similarly to the previous patients, since there were associated risk factors. In the patients pre-treated with Alveogyl, it was necessary to apply six-time HA+octenidine, which proves that Alveogyl reduces the effectiveness of treatment with HA+octenidine. Similarly, the smoker patient required seven applications of HA+octenidine. The effect of tabacco is mainly in the vasoconstriction of the mucosal blood vessels and the local involvement of the inflammatory reaction. Furthermore, the suction process can be a determining factor in the clot disintegration process. In summary, the study conducted by this group shows that factors such as smoking and previous treatment with Alveogyl can decrease the effectiveness of treatment with the HA+octenidine device [164].

### 5.9. Bladder Inflammation

Interstitial cystitis and/or bladder pain syndrome (IC/BPS) are characterized by discomfort, abdominal pain, and pain in the pelvic region. These conditions induce a strong inflammatory response, which begins with damage to the epithelial barrier. The epithelial layer of the bladder is protected by GAGs, such as HA [165]. In inflammatory diseases of the bladder, defects occur at the level of this lining, which protects the submucosa of the bladder from urinary solutes [165,166]. When it is damaged, it becomes permeable, allowing toxic substances to penetrate the bladder wall, causing a cascade of inflammatory responses that result in additional pain and damage to the urothelium [166]. These defects are due to the continued loss of GAGs in the urinary epithelium [165].

Stellavato et al., studied the in vitro efficacy of HA and chondroitin sulphate (CS), alone or in combination (HA/CS), in a model of bladder cells pre-treated with TNF-α to mimic IC/BPS. The results show that the inflammation was reduced in the presence of HA, CS and the combination of both (HA/CS). All the analysed biological markers (IL-6, IL-8, NF-кB, zonulin (ZO-1, a permeability regulator), and hBD-2) show that both CS and HA have the ability to modulate cells to a more physiological state, improving cell-cell communication and helping to restore the bladder barrier. HA/CS reduced the expression of NF-кB, and increased the expression of hBD-2 and the levels of ZO-1, which led to a reduction in the permeability of the endothelium and, therefore, the renewal of the epithelial tissue to a more physiological condition [165].

Using cystitis as a model, Shainer et al., studied the effect of treatment with intravesical HA in a mouse model with hydrochloric acid-induced cystitis (HCl). The data show that the severity of inflammation decreased in the HA-treated group compared to the control. It was also found that the integrity of the bladder mucosa is preserved. A decrease was found in mast cell counts and IL-6 levels [167]. Similarly, Yildiz et al., evaluated the effect of intravesical HA on cystitis induced by *Escherichia coli* (*E. coli*) in rats. The results show that, during bacterial cystitis, the activity of tissue myeloperoxidase increased, but the activities of superoxide dismutase and catalase decreased. HA treatment can reverse these changes. Regarding the uroepithelium healing, HA treatment seems to decrease the increased threshold of contraction, reduce oxidative stress and limit the infiltration of inflammatory cells. In comparison, in the treatment with gentamicin, the infiltration of inflammatory cells was reduced, but there was not improvement in oxidative degeneration, which shows that HA may have an important role in the treatment of bacterial cystitis [168].

Rooney et al., also using cystitis as a basis, created a biphasic system by combining cross-linked HA and naïve HA (cHA:HA) solution to decrease inflammation and permeability. HTB-2 cells were used to monitor the metabolic activity of different concentrations of HA (1, 3, 9 and 15 mg/mL). The results show that, in the control groups, the metabolic activity is not affected. However, in the presence of damage to the cell barrier (caused by protamine sulfate (PS)), the higher concentration significantly increased metabolic activity. Thus, to avoid effects that are dependent on HA concentration, only concentrations of 1 and 3 mg/mL were tested in the biphasic system. Inflammation in the urothelium barrier (in T84 cells grown on transwell permeable membrane) was simulated by the addition of TNF-α, and PS was used to damage the barrier. Cross-linked HA alone, at 1 mg/mL in PS-treated cells, increased the levels of MCP-1 and IL-6, while a concentration of 3 mg/mL only increases levels of IL-6. Naïve HA at a 1 mg/mL concentration increased IL-6 and MCP-1 in inflamed TNF-α; at a concentration of 3 mg/mL, IL-6 levels increase when cells are treated with PS and TNF-α. The biphasic system in the proportion of 1:1 does not increase the levels of IL-6, IL-8 or MCP-1 at concentrations of 1 and 3 mg/mL. In these conditions, the biphasic system decreases the permeability of the cell monolayer (assessed by the value of transepithelial electrical resistance (TEER)), making it a promising approach for IC treatment [169].

### 5.10. Renal Inflammation

Acute kidney injury (AKI) is a serious kidney disease characterized by rapid loss of kidney function, leading to the accumulation of metabolic waste, electrolyte, and body fluid imbalances. The pathophysiology of this disease is associated with complicated mechanisms, such as the cause of oxidative stress, vascular damage, and inflammation. AKI is characterized, at the pathological level, by severe and even lethal damage to the renal tubules, leading to tubular dysfunction and cell death [170].

Despite presenting a low solubility in water and, therefore, a low bioavailability in biological systems, curcumin (CUR) is a polyphenol with antioxidant and anti-inflammatory properties, with studies showing that CUR is able to protect against the kidney damage caused by ischemia-reperfusion. To overcome these limitations, a polymeric HA-CUR (HA-CUR) prodrug directed to epithelial cells was developed. Studies have shown that the solubility of HA-CUR was increased compared to free CUR. Cell internalization studies show that HA-CUR was preferentially internalized by tubular epithelial cells compared to free CUR. In terms of biodistribution, the results also show an increase in the accumulation of HA-CUR in the kidneys when compared to free CUR. The levels of pro-inflammatory cytokines (TNF-α and IL-6) and oxidative stress (superoxide dismutase (SOD), glutathione (GSH) and malondialdehyde (MDA)) were reduced in the presence of CUR and HA-CUR, with a better therapeutic efficacy in the presence of HA-CUR [170].

### 5.11. Cardiac Inflammation

Almost any anomaly at the heart level can initiate an inflammatory response and even systemic inflammation can trigger various inflammatory pathways within the heart tissue. Acute cardiac inflammation can result in a rapid decline in heart function, while chronic inflammation causes progressive structural damage, leading to the development of cardiac fibrosis [65]. In the heart, HA is involved in physiological functions, such as cardiac development during embryogenesis, and in pathological conditions, including atherosclerosis and myocardial infarction [171].

Myocardial infarction (MI) appears when a coronary artery becomes blocked, leading to insufficient blood supply to the myocardium in the affected area. This leads to a reduction in oxygen distribution, nutrient and metabolic waste input and output. The repair mechanism of IM is a complex process, with different types of changes in cellular and extracellular components. Wang et al., synthesized HA oligosaccharides (o-HA) with 6–10 oligosaccharides through enzymatic degradation. Studies carried out on an MI model (subacute model of rat) show that o-HA can reduce the size of the infarction and the apoptosis of cardiomyocytes in the MI region, as well as promote myocardial angiogenesis and the reconstruction of myocardial function. It was also shown that o-HA improved the polarization of M2 macrophages and removed the inflammatory response caused by neutrophils to accelerate the reconstruction of myocardial function [172].

In a similar approach, Yoon and colleagues developed an HA-based injectable hydrogel for MI regeneration and functional recovery of the heart. The hydrogel, when injected into the epicardium of the affected area (subacute model of rat, Sprague-Dawley male rats), showed, an increase in wall thickness after 4 weeks, as well as a decrease in the infarcted area. The number of arterioles and capillaries increased, and the apoptotic index decreased, when compared to the IM control group. The measurement of cardiac function indicated that hydrogel injection significantly facilitated functional recovery compared to the MI control. Due to the hygroscopic properties of HA, the hydrogel has a high expansion rate, which can lead to a change in the microenvironment in vivo. As this expansion occurs, fluids containing cytokines and other small molecules are absorbed, which in turn leads to decreased inflammation [173].

## 6. Conclusions and Future Outlooks

HA has an excellent potential for a wide range of applications, which comprise much more than the facial treatments with which it is typically associated. Its wide applicability is due to its distinctive biophysical properties, which give it enormous potential. As previously reported, the fact that HA is found in virtually all cell types is indicative of its biological advantages, ranging from its mechanical and swelling properties to lubrification, tissue regeneration and hydration.

A relevant aspect for all these biological properties is the cell surface receptors with which HA can interact, including the CD44 receptor, which is widely expressed in activated macrophages (which play a central role in the inflammatory process). It is the interaction with these receptors that enables the targeted delivery to target locations, resulting in greater cellular uptake and, therefore, beneficial results in reducing inflammation.

Effectively, what is verified is that HA has been the subject of numerous studies in several pathologies. However, if the focus of HA applications is only inflammatory pathologies, especially OA and RA, it is clear that a great deal of effort has been devoted to the use of this molecule in the search for alternatives to conventional treatments.

Although it is clear that HA’s biodegradability and biocompatibility contribute to its widespread use, either alone, in conjunction with other active ingredients, or in the formulation of nanoparticles, more studies should be carried out with the aim of obtaining prolonged circulation and a longer time in situ. A more detailed understanding of the degradation mechanisms of HA will also be essential to optimizing and improving the biomedical applications of this polymer. The development of methods that allow for a simpler administration (such as oral administration) of HA-based products would also be an asset. Similarly, studying how HA acts in extra-articular inflammatory pathologies will also be an essential step towards getting the most out of this powerful molecule.

The major complications associated with the delivery of HA are due to its relatively short half-life in biological fluids. This means that it is often necessary to resort to crosslinking techniques, which can lead to greater toxicity compared to “free” HA. The viscosity of HA solutions can also raise some questions, as this can hamper the administration or effectiveness of the developed models. However, the studies reviewed here show promising results for the use of HA in the treatment of inflammatory pathologies.

## Figures and Tables

**Table 1:** Study summaries, with HA, for the treatment of OA.

Formulation		Studies	Administration Route	Main Results	Ref.
HA-Clx	Liposomes	In vitro and in vivo	IA	Pain control, and cartilage protection	[80]
HA/PEG/KGN	Micelles	In vitro and in vivo	IA	Suppression of OA progression	[81]
SASP/HA	Conjugate	In vitro and in vivo	IA	Inhibition of cartilage destruction, and OA progression	[82]
Disks and injectable thermosensitive gels with PD and TGF-β3	Scaffolds	In vitro and in vivo	IA	Decrease in osteoarthritis score, and global score	[83]
HA-MTX	Conjugate	In vivo	IA	Reduction of swelling, and inflammation	[84]
DK226	Conjugate	In vivo	IA	Reduction of swelling, and inflammation	[85]
FidHycarn	Conjugate	In vivo	Oral	Reduced levels of cytokines, chemokines, and oxidative damage	[86]
HA/CS-CrmA	Polymeric nanoparticles	In vitro		Reduction of cartilage damage and inflammation	[87]
pH-responsive nanoparticles with NH_4_CO_3_ and HA	Polymeric nanoparticles	In vitro and in vivo	IA	Reduction disease progression	[88]
HA-loaded PLGA particles	Hydrogel	In vitro and in vivo	IA	Inhibition of inflammation	[89]
HMWHA		In vivo	IA	Restoration of normal joint structure	[90]
HABP2-8-arm PEG-COLBP	Peptide-polymer platform	In vivo	IA	Reduced levels of pro-inflammatory cytokines	[91]
HTG-E	Hydrogel	In vitro and in vivo	IA	Induction of chondrogenic regeneration and loss of minimized cartilage	[92]
HAMPC	Lubricated polymer	In vitro		Lubrication capacity and reduction of inflammation	[93]
pNIPAM	Hydrogel	In vitro and in vivo	IA	Lubrication capacity and ability of intelligently releasing	[94]
PRP+HA		In vivo	IA	Inhibition of inflammation	[95]
HA-Lipo-DIC/DEX	Liposomes	In vitro and in vivo	IA	Inhibition of inflammation and increased cell numbers	[96]
Celecoxib-HA	Nanocapsules	In vitro and in vivo	IA	Inhibition of inflammation and	[97]
HA-gel+magnoflorine		In vitro and in vivo	IA	Protects effect on the cartilage matrix	[98]
In situ crosslinking N-chitosan, ADH and HA-ALD	Hydrogel	In vitro and in vivo	IA	Prevention of cartilage destruction and pain relief	[99]

**Table 2:** Study summaries with HA, for the treatment of RA.

Formulation		Studies	Administration Route	Main Results	Ref.
HA-SLN/PD	Lipid nanoparticles	In vitro and in vivo	Intravenous	Bone preservation and reduced levels of pro-inflammatory cytokines	[101]
pH-sensitive liposomes with PDP	Liposomes	In vitro		Increased bioavailability and effectiveness of PDP	[102]
HAPNPs/Dex	Polymeric nanoparticles	In vitro and in vivo	IA	Reduction in inflammatory cell infiltration, bone damage and cartilage	[66]
DNPs	Polymeric nanoparticles	In vivo	Systemic	Reduced levels of pro-inflammatory cytokines	[103]
MP-HANPs	Polymeric nanoparticles	In vitro and in vivo	Systemic	Accumulation at the inflammation site	[104]
HA/Cur	Nanomicelles	In vitro and in vivo	IA	Reduction of inflammation and protection of cartilage	[105]
FidHycarn	Conjugate	In vivo	oral	Reduced levels of pro-inflammatory cytokines and chemokines	[106]
Chitosan nanogels for the delivery of PS	Nanogels	In vitro and in vivo	IA	PS retention in the joint and inflammation reduction	[107]
HA-MTX	Conjugate	In vitro and in vivo	Intravenous	Reduction of inflammatory cytokine levels and cartilage damage	[108]
Flare-responsive TG-18 and TA delivery system	Hydrogel	In vitro and in vivo	IA	Reduced the arthritis activity	[109]
HYA-HAMT-NPs	Polymeric nanoparticles	In vitro and in vivo	IA	Preventing disease progression and promoting joint regeneration	[110]
HA-AuNCs/T/P	Gold nanocages	In vivo	Intravenous	Bone and cartilage preservation	[111]

**Table 3:** Study summaries where HA is used for wound-healing.

Formulation		Studies	Administration Route	Main Results	Ref.
HA, MH and AG		In vivo	Topic	Reduced healing time with the use of HA	[133]
BHA	*N*-butyrylated LMW-HA	In vitro and in vivo	Topic	Reduction in pro-inflammatory cytokines levels; increased expression of growth factors	[136]
HA-POL	Hydrogel	In vitro and in vivo	Topic	Increase in protein accumulation; improvement in tissue repair	[137]
photo-responsive supramolecular hydrogels for EGF delivery	Hydrogel	In vitro and in vivo	Topic	Granulation tissue formation, and growth of neoepidermis	[138]
FHHA-S/Fe	Hydrogel	In vitro and in vivo	Topic	Decreased average wound area	[139]

**Table 4:** Study summaries using with HA for the treatment of inflammatory bowel disease.

Formulation		Studies	Administration Route	Main Results	Ref.
IBD98-M	Conjugate	Ex vivo and in vivo	Injected into the ligated area of the distal colon	Promotion of healing of the intestinal mucosa; reduction in intestinal inflammation	[140]
HANPs with BDS	Polymeric Nanoparticles	In vitro		Inflammation reduction	[141]
HA-KPV-NPs	Polymeric nanoparticles	In vitro and in vivo	Oral	Inflammation relief; mucosal healing; combination with hydrogel exhibits increased ability to prevent mucosal damage	[145]
HMWHA		In vitro and in vivo	Local application	Improvement in tissue regeneration	[146]
HMWHA		In vivo	Systemic	Relief of UC symptoms; increased expression of COX-2 and PGE_2_	[147]
HABN	Conjugate	In vivo	Oral	Colon epithelium barrier restored	[148]
